# Children’s Active Commuting to School: Current Knowledge and Future Directions

**Published:** 2008-06-15

**Authors:** Kirsten K Davison, Jessica L. Werder, Catherine T Lawson

**Affiliations:** Department of Health Policy, Management and Behavior, University at Albany, SUNY; Department of Health Policy, Management and Behavior, University at Albany, SUNY, Rensselaer, New York; Department of Geography and Planning, University at Albany, SUNY, Rensselaer, New York

## Abstract

**Introduction:**

Driven largely by international declines in rates of walking and bicycling to school and the noted health benefits of physical activity for children, research on children's active commuting to school has expanded rapidly during the past 5 years. We summarize research on predictors and health consequences of active commuting to school and outline and evaluate programs specific to children's walking and bicycling to school.

**Methods:**

Literature on children's active commuting to school published before June 2007 was compiled by searching PubMed, PsycINFO, and the National Transportation Library databases; conducting Internet searches on program-based activities; and reviewing relevant transportation journals published during the last 4 years.

**Results:**

Children who walk or bicycle to school have higher daily levels of physical activity and better cardiovascular fitness than do children who do not actively commute to school. A wide range of predictors of children's active commuting behaviors was identified, including demographic factors, individual and family factors, school factors (including the immediate area surrounding schools), and social and physical environmental factors. Safe Routes to School and the Walking School Bus are 2 public health efforts that promote walking and bicycling to school. Although evaluations of these programs are limited, evidence exists that these activities are viewed positively by key stakeholders and have positive effects on children's active commuting to school.

**Conclusion:**

Future efforts to promote walking and bicycling to school will be facilitated by building on current research, combining the strengths of scientific rigor with the predesign and postdesign provided by intervention activities, and disseminating results broadly and rapidly.

## Introduction

Rates of walking and bicycling to school, or active commuting, have declined precipitously during the past 30 years. According to the National Household Travel Survey, less than 16% of students aged 5 to 15 years walked or biked to school in 2001 (1). In contrast, 48% of children in this age range walked or biked to school in 1969 (2). Furthermore, data from the 1999 HealthStyles Survey indicate that only 31% of children who live within 1 mile of school actively commute to school (3). Rates of walking and bicycling to school have decreased against a backdrop of declining levels of physical activity and increasing prevalence of overweight among youth (4-6), which suggests that these trends are linked. Driven largely by international declines in rates of active commuting to school and the noted health benefits of physical activity for children (7), research on children's active commuting behaviors has expanded rapidly during the past 5 years. This accumulating body of research provides insight into predictors of children's active commuting to school and their associated health consequences. In this article, we summarize the results of pertinent studies and programmatic activities specific to children's active commuting to school in an effort to guide public health efforts to promote walking and bicycling to school. Our 3 goals are to 1) examine research on the health consequences of active commuting to school, 2) summarize pertinent studies on predictors of children's active commuting to school, and 3) outline and evaluate programs specific to children's active commuting to school. We conclude by discussing methodological problems in research and suggesting future directions.

## Methods

To identify research on the predictors and health consequences of walking or bicycling to school (goals 1 and 2 outlined above), we searched published articles by using PubMed and PsycINFO electronic databases. Search terms employed were *children*, *school*, *walk*, *bike*, *cycle*, *active* *commute*, *active* *transport*, *school* *journey*, and *school* *siting*. Potential articles were assessed against the following inclusion criteria: 1) the study sample was composed of children younger than 18 years, 2) the study measured children's active commuting behaviors and predictors or consequences of these behaviors, and 3) the study was published in English in a peer-reviewed journal before June 2007. We also reviewed the bibliographies of articles that meet inclusion criteria to identify referenced sources that we may have missed.

Given that much of the information about programs is located online and in reports and documents from the transportation literature, a more elaborate method was necessary to achieve our third goal. We searched for studies on the Safe Routes to School and the Walking School Bus by using PubMed. We located materials from the transportation research and practice community by searching the National Transportation Library (http://ntl.bts.gov) and the recently enhanced State DOT Google Search Engine and by reviewing relevant transportation journals (e.g., *Transport Policy*) published during the last 4 years.

## Relationship Between Active Commuting and Health

Information about the relationship between children's active commuting and health illustrates the public health significance of this topic and provides a backdrop for the examination of predictors of active commuting. Key health indicators that have been examined in the literature include physical activity, body mass index (BMI), and cardiovascular fitness.

Research shows that children and adolescents who walk or bicycle to school have higher daily levels of physical activity and are more likely to meet physical activity recommendations than are youth who travel to school by car or bus. These effects are noted when both self-report (8-10) and objective (11-16) measures of physical activity are used and are evident among elementary- (8,10,12,13,15), middle- (11,16), and high-school–aged (9,14) children. Although most studies show a significant, positive association between active commuting to school and level of physical activity, 2 studies from those identified failed to identify a significant effect (17,18).

Given the limitations of self-report in assessing physical activity (19), the most credible source of information about the link between children's active commuting to school and their physical activity levels is research using objective measures of physical activity (e.g., activity monitors, also known as accelerometers). Data from these studies indicate that children who use active forms of transport to school accumulate approximately 20 additional minutes of moderate to vigorous physical activity (MVPA) per day on weekdays (8,11,13,15) and expend 33.2 to 44.2 kcal more per day than do youth who are driven to school (14). Links between active commuting and physical activity may be stronger for boys than for girls (10,13). For example, boys who actively commuted to school accumulated 45 more minutes of MVPA per day than did boys who were driven to school, whereas active commuting girls accumulated only 4 more minutes of MVPA than did girls who were driven to school (13). Although none of the studies directly examined the temporal order of the association between active commuting and levels of physical activity, differences in physical activity levels for active and nonactive commuters generally were limited to weekdays (13,15). This finding suggests that differences in physical activity by transport mode are not simply due to the fact that more active children choose active forms of transport to school.

A small number of studies have assessed associations between active commuting and children's BMI and cardiovascular fitness. According to data from the European Youth Heart Study, children and adolescents who cycled to school were nearly 5 times as likely to be in the top quartile for fitness than were youth who walked or used motorized forms of transport (20). In contrast to research focusing on children's physical activity and fitness, little support exists for an association between active commuting and children's BMI (8,14,17,21). In one of the few longitudinal studies, no association was found between active commuting to school and children's change in BMI over a 2-year period (17). Furthermore, in contrast to what was anticipated, BMI of active commuters increased more during a 5-month period than did BMI of nonactive commuters (8). This effect was largely driven by increases in BMI among overweight children. In the only study that identified a significant effect in the anticipated direction, middle-school–aged youth who were at risk for overweight (≥85th and <95th BMI percentile) were less likely than their nonoverweight counterparts to walk or cycle to school; no effects were found for overweight youth (≥95th BMI percentile) or for high-school–aged youth (22).

Despite weak evidence linking active commuting to reduced BMI, research indicates that possible health benefits of active commuting to school include higher rates of physical activity and higher cardiovascular fitness among youth, which are linked with reduced risk for coronary heart disease, stroke, cardiovascular disease, and cancer (23). Possible health benefits for children of walking and bicycling to school illustrate the need to further examine ways to promote active commuting in this population. The first step in this process is to identify factors that increase the likelihood that children will walk or bicycle to school.

## Predictors of Active Commuting to School

Research on predictors of children's active commuting to school has expanded rapidly during the past 5 years. Key categories of predictors that have emerged from the literature include characteristics of the individual and family, school, and social and physical environments. Research on each of these predictors is presented below. All studies outlined used a cross-sectional design, so discussing the direction of these associations is not possible.

### Individual and family characteristics

Research on individual characteristics generally has focused on differences in rates of active commuting among demographic groups, including groups that differ by race/ethnicity and socioeconomic status (SES). In general, research shows that Hispanic and African American children are more likely than white children to actively commute to school. In a study of 34 schools in California, rates of active commuting were higher in schools with a higher proportion of Hispanic students and lower in schools with a higher proportion of white students (24). Data from North Carolina suggest a similar pattern, with African American students walking and biking to school more regularly than white students (22). Similarly, research suggests that children from low SES backgrounds are more likely than children from high SES backgrounds to actively commute to school. This effect is noted using school-level SES measures (e.g., percentage of students on welfare) (24), neighborhood-level SES measures (25), and measures of household income and home ownership (26,27). Although most studies indicate that children from low SES backgrounds are more likely to actively commute, no association was found between school-level SES and active commuting to school (28). Furthermore, 3 out of 4 studies found no association between family ownership of a car (an indirect measure of SES) and children's mode of transport to school once other demographic factors and distance were taken into consideration (26,27,29,30).

Along with general demographic factors, decisions about children's transport to school often reflect the needs and characteristics of children and their parents. Child characteristics that have been examined include sex, age, and attitudes about physical activity. Research shows that boys are more likely than girls to actively commute to school (9,14,17,22,25), with one study showing boys almost twice as likely as girls to walk or bicycle to school (25). This pattern is noted both in the United States (22) and internationally (9,14,25). Higher rates of walking to school among boys may reflect social tendencies of parents to be more protective of girls and to place greater restrictions on girls' independent mobility. Such differences also may reflect sex differences in general levels of physical activity (31).

Some studies indicate that older children are more likely than younger children to actively commute to school (27,30,32), whereas other studies show the opposite pattern (9,22,33). This inconsistent pattern may reflect the possibility that the relationship between age and active commuting is not linear. Children's age-related gains in independent mobility may be linked with higher rates of active commuting until they acquire drivers' licenses and begin to drive themselves to school. Furthermore, cross-study differences in age-related changes in active commuting may reflect regional differences in the size and location of middle and high schools, such that age-related gains in mobility may be overridden in districts where schools are not accessible by active modes of transportation. Finally, no associations have been identified between children's attitudes about physical activity, including children's eagerness to walk (30) or their enjoyment of physical activity (34), and their active commuting patterns.

As with any child behavior, commuting to school is influenced by parent and family attributes and circumstances. Evidence suggests that children are less likely to actively commute when their parents work (34) and when the active commuting interferes with parents' work schedules (26) and children's after-school commitments (34). Conversely, children are more likely to actively commute if their parents actively commuted to school (30,34) and currently actively commute to work (30). Furthermore, children are more likely to actively commute when parents value physical activity (30,34) and the social interactions that take place among children during the commute to school (26). Other issues raised by parents, which are discussed later in greater detail, include perceived neighborhood safety, traffic safety, distance to school, and weather.

### School characteristics

Characteristics of the school, including school administration and location, may influence rates of active commuting because of the accessibility of the school from the communities in which children live. Regarding school administration, students who attend public schools are more likely than students who attend private schools to actively commute to school (30). Similarly, children who attend independent schools (i.e., a subset of private schools) are less likely than are children who attend public schools and religiously affiliated schools to walk to school (27). Although these effects may have reflected differences in travel distance to school — with students attending private or independent schools living farther from school than students attending public schools — or differences in car ownership, Merom and colleagues (30) ruled out these explanations. Thus, explanations for differences in rates of active commuting by school type are not clear.

School and community factors associated with school location (referred to as *school siting*), such as distance to school, population density in the immediate area of a school, and school enrollment levels, have been consistently linked with active commuting rates. More than 55% of parents reported that distance is a barrier to their children's active commuting to school (3,35), making it the most frequently reported barrier (3,8,35) and the strongest predictor of children's walking and biking to school (25,26,30,34,36). Australian children were more than 5 times more likely to walk or bicycle to school at least once per week if they lived within 800 m (approximately 0.5 miles) of their school than were children who lived farther away (29). In the United States, children who lived within 1 mile of school were more than 3 times as likely to walk or bicycle to school than children who lived greater distances from school (26). Lower school enrollment and greater population density within 0.5 miles of the school were associated with higher rates of walking and biking to school (24). Both of these indicators probably reflect school siting, because larger schools (i.e., satellite schools) are located outside of general residential areas (with low population density) and draw on a larger pool of students from a broader geographic area.

### Community and environmental characteristics

Beyond schools and families, many community and environmental factors have been examined as predictors of children's commuting patterns, including elements of the physical (e.g., transport infrastructure, weather) and social (e.g., crime, social norms) environment. With the exception of weather, all of the environmental factors examined are modifiable and therefore amenable to change.

Physical environmental factors that may influence children's mode of transport to school include road and sidewalk infrastructure, traffic safety, accessibility of public transportation, urban vs rural location, and weather conditions. Research examining characteristics of the physical environment suggests that children are more likely to walk or bicycle to school when the route to school is direct (29), navigation of steep roads is minimal (29), and neighborhoods in which children live are deemed "walkable" (as measured by residential density, retail floor area ratio, intersection density, and land use mix) (26,37). In contrast to characteristics of road and sidewalk infrastructure, most studies find no association between perceived traffic safety and children's active commuting. Although in one study parents' concerns about traffic safety were associated with lower rates of active commuting among children (26), 3 additional studies found no relationship between perceived road or pedestrian safety and children's mode of transport to school (27,29,37). Furthermore, access to public transport was not associated with children's walking and bicycling to school (29). For residential location, evidence suggests that children who live in rural areas are less likely than children who live in urban areas to actively commute to school (9,36), which may be related to less infrastructure for walking, longer commuting routes, and decreased access to public transportation. Finally, in contrast to studies that examined modifiable environmental factors, no association was found between weather and directly observed rates of active commuting (28). Data for this study, however, were collected in South Carolina during milder months of the year (28). Few studies have examined the effects of extreme cold and heat on children's commuting patterns.

Social environmental factors focus on interactions among individuals and encompass crime and social norms. Little research has focused on social environmental predictors of children's active commuting to school. Mixed results have been identified for studies assessing perceived crime and safety. Similar to effects noted for traffic safety, children were more likely to walk or bicycle to school when parents perceived the neighborhood as safe and when a greater percentage of houses within 0.25 miles from the school had windows facing the street — a measure of "eyes on the street" (26). In contrast, no relationship was found between concern about crime or strangers and children's active commuting to school (27,29,37). More consistent results are noted for perceived social norms. This research shows that children are more likely to walk or bicycle to school when parents perceive that other children in the area actively commute (29) and when other family members agree with the decision to allow a child to walk or bicycle to school (26).

Although most research has assessed the role of specific physical or social environmental characteristics, many studies have compared the contribution of objectively measured characteristics of the physical environment, referred to as *urban form variables* (e.g., mixed land use, street connectivity), and parents' reports of social and physical environmental factors, with the prediction of children's active commuting behaviors. Findings from these studies indicate that parents' perception of the environment is a stronger predictor of children's active commuting than are urban form variables (26,37), which suggests that any changes to the physical environment are unlikely to affect children's active commuting patterns unless parents' concerns and attitudes also are addressed.

### Summary and conclusions

Many factors, reflecting characteristics of children and families, schools, communities, and the environment, have been examined as potential predictors of children's active commuting to school. Regarding individual and family characteristics, research suggests Hispanic and African American children and children from low SES backgrounds are more likely to actively commute to school than are white children and children from high SES backgrounds. These findings do not appear to result from differences in family car ownership but may reflect differences in residential location (30). Research also indicates that, although boys are more likely than girls to actively commute to school, child characteristics such as age and enjoyment of walking and physical activity are not consistently related to active commuting rates. These findings suggest that child characteristics do not drive parents' decisions about children's mode of transport to school. Parent and family characteristics, however, are consistently linked with children's active commuting patterns. Children are more likely to walk and bicycle to school when the active commuting does not interfere with parents' work schedules or children's after-school commitments, when parents actively commuted to school as children or currently walk or bicycle to work, and when parents value physical activity and the accompanying social interactions for their children.

Regarding school characteristics, distance to school is the most readily identified barrier to children's active commuting and is the strongest predictor of their mode of transport to school, with larger distances associated with lower rates of active commuting. Furthermore, research suggests that children are more likely to actively commute when the immediate areas surrounding schools are more densely populated and when school enrollments are lower. All of these factors reflect school siting.

For environmental characteristics, children are more likely to walk or bicycle to school when they live in urban neighborhoods and when road and sidewalk infrastructure (e.g., presence of controlled intersections, a direct route to school, few hills) and social norms support active commuting. Inconsistent findings were noted for perceived safety, including traffic safety, perceived crime, and "stranger danger," and no effects were observed for weather or the presence of public transportation. Finally, when objectively measured environmental characteristics are compared with parents' perception of environmental attributes, research indicates that parents' perception of the environment is a stronger predictor of children's active commuting patterns.

### Limitations of research

Although such conclusions can help guide active commuting programs, limitations of the research need to be considered. All studies on predictors of active commuting used a cross-sectional design. Therefore, whether each predictor leads to or results from children's active commuting is unclear. For example, parents who consider walking or bicycling to school to be important and whose children have a history of active commuting may choose to live in areas that have the necessary infrastructure to support walking. Additional limitations include the lack of standardized definition and measurement of active commuting. Some studies used parents' estimates of their children's frequency of walking or bicycling to school. Other studies relied on children's reports and included methods such as taking a "hand count" of those who walked or bicycled to school on a particular day. Still others directly observed pedestrian traffic around schools. Another weakness of this research is that no clear theory- or evidence-based rationale exists for the identification and examination of possible predictors of active commuting to school. The issue of poorly conceptualized predictor variables is highlighted by a large number of studies that found significant associations between commuting patterns and the variables classified as "other," indicating that important variables may be at play that have yet to be addressed and that research techniques, such as the use of focus groups, may be an important first step in determining which variables are important for further analysis. Finally, many of the predictor variables were assessed only in a single study, thereby limiting the conclusions. Many potentially important factors, such as bussing policies and school policies (e.g., age or grade regulations on walking and bicycling to school, the presence of crossing guards and bicycle racks), were notably absent in current research. Additional research is needed, and the list of potential predictors examined in the future needs to be expanded on the basis of the theory of key informant interviews.

## The Role of Programmatic Strategies

In tandem with the noted increase in research-based activities, national and international programmatic activities supporting children's active commuting have expanded in recent years (Box). These activities overlap and often are implemented simultaneously. For example, establishing a Walking School Bus has been encouraged as part of International Walk to School Month. The evidence base was limited when these programs were first established. Therefore, most programs were founded on the basis of what was thought to impact children's active commuting patterns at the time. However, the implementation and evaluation of such programs complements research efforts and provides additional insight into predictors of children's active commuting patterns and targets for future intervention efforts. The Safe Routes to School and the Walking School Bus programs are outlined in greater detail below to illustrate these points. We focused on these 2 programs, because they are national programs and have more complete evaluation data.

- KidsWalk-to-School (www.cdc.gov/nccdphp/dnpa/kidswalk)
- Ride2School (www.bv.com.au/join-us/125)
- Safe Routes to School (www.saferoutesinfo.org)
- Walking School Bus (www.walkingschoolbus.org)
- International Walk to School Month (www.iwalktoschool.org)

In the United States, state-level efforts to promote safe routes to school emerged during the 1990s and culminated in 2005 with the federally funded Safe Routes to School (SRTS) program, founded under the umbrella of the Safe, Accountable, Flexible, Efficient Transportation Equity Act: A Legacy for Users (SAFETEA-LU) (38). Although this type of program was new for the United States, it grew from existing international efforts to organize and promote active commuting (39-41). The goals of the SRTS program are to 1) enable and encourage children, including those with disabilities, to walk and bicycle to school; 2) make walking and bicycling to school a safe and more appealing transportation alternative; and 3) facilitate the planning, development, and implementation of projects and activities that will improve and reduce traffic, fuel consumption, and air pollution in the vicinity of schools (38). The SRTS program makes funds available to states through the Department of Transportation for a broad range of activities, including infrastructure-based (e.g., improvements in roads and sidewalks) and noninfrastructure-based (e.g., education, enforcement) activities, as well as coordinated dissemination of information through a state SRTS coordinator, a national SRTS clearinghouse, and a SRTS task force.

Although few evaluations of SRTS programs have been published, results are available from 3 such programs in California. Staunton et al relay the success story of Marin County, California, in implementing a program to increase the number of children walking to school while reducing the number of car trips to school (42). Incorporating elements of classroom education, walking and biking days, mapping of routes, and walking trains and newsletters, the program resulted in a 64% increase in the number of children walking to school (42). Using case studies to evaluate specific SRTS projects for their effects on active commuting, Boarnet et al (43) concluded that sidewalk gap closure projects (i.e., connecting previously disconnected sections of sidewalk) and improvements in traffic signals (e.g., replacing a 4-way stop sign with traffic lights) were associated with increased rates of walking and increased pedestrian safety among children. Limited effects were noted for appropriate sidewalk signage, improvements in crosswalk signals (e.g., flashing warning light systems at crosswalks), and the presence of bike lanes. In a similar analysis, the presence of SRTS projects on students' behavioral patterns was assessed (44). Findings suggested that children who passed an SRTS project on their route to school were more likely to increase their personal active commuting patterns. However, the authors noted an overall decrease in walking and bicycling rates after completion of the projects, potentially because of the pattern of increased driving during project construction that was difficult to break after construction was complete (44).

As a national program, SRTS is being implemented throughout the United States. Although these programs and projects have yet to be evaluated comprehensively, the National Center for Safe Routes to School (www.saferoutesinfo.org) is currently tracking the spending patterns by state programs participating in the federal funding strategy (45). As of March 31, 2007, all 50 states and the District of Columbia had demonstrated some level of participation in the SRTS program. Nineteen states have spent approximately $24.3 million for SRTS on infrastructure and noninfrastructure (i.e., education, training) projects.

A second public health effort to promote active commuting to school is the Walking School Bus (WSB) program. In its simplest form, the WSB is a voluntary program that requires one or more adults to escort small groups of children, on foot or bicycle, to and from school each day. The group establishes a meeting point for participants, referred to as a "bus stop," and proceeds as a group in active commute mode. Although little formal evaluation is available on this grassroots strategy, many small-scale qualitative studies from New Zealand provide insight into the benefits of the WSB program and factors affecting its sustainability. Benefits of the WSB program, as reported by parents and other stakeholders, include eliminating the hassle of driving to school and finding a parking spot, knowing that children are safe, providing an opportunity for children to socialize with other children, gaining independence through walking, increasing health benefits through walking, raising awareness of children's road safety, and increasing civic participation (46,47). In one of the few long-term follow-up studies, Kingham and Ussher (48) examined factors impacting the sustainability of the WSB program in Christchurch, New Zealand, from its initiation in September 2000 to its follow-up in April 2003. During this time, the number of routes declined 54%, with few routes surviving beyond a year, and the average number of children on each route declined from 9 to 7.7. Barriers to the sustainability of the program reported by parents and program organizers included a lack of parent volunteers, weather (i.e., confusion about whether the WSB would operate during inclement weather), road safety, and lack of communication between schools and parent organizers. Thus, although parents and stakeholders readily identified the benefits of the WSB, the ability to sustain the program was compromised by many factors, ranging from organizational issues to road infrastructure.

In summary, both the SRTS and WSB programs are designed to promote active commuting to school. SRTS is a broad-scale effort that includes changes in transport infrastructure and school policies, as well as educational activities within and outside of school. SRTS programs now operate in all 50 states and the District of Columbia. Although these programs have a broad reach, they do not lend themselves readily to evaluation because of the breadth of changes, lack of standardized protocols, absence of control schools, and lack of information about children's behavior before program implementation. Evaluation data suggest that improvements in sidewalk infrastructure and traffic calming measures may be more effective than improvements in sidewalk signage and the installation of crossing signals and bike lanes. Furthermore, programs that couple infrastructure changes with classroom activities and parent involvement may be most effective. This last finding is supported by research highlighting the role of parents as gatekeepers to children's ability to walk or bicycle to school.

In contrast to the broad-scale approach of SRTS, the WSB program is a smaller-scale grassroots effort that can be incorporated into SRTS programs. The WSB program focuses on mobilizing groups of parents and stakeholders to create supervised walking routes for children and in general does not address structural changes to promote safe walking and bicycling. Although WSB programs are more focused — and therefore more amenable to evaluation — than SRTS, they generally are organized at the local level with few or no resources. Consequently, evaluation and dissemination of findings are not mandated or of central interest in these programs. Although parents and stakeholders readily identify benefits, WSB programs are difficult to sustain because of issues such as a lack of parent volunteers and unsafe road conditions.

The SRTS and WSB programs were established when little information had been published about predictors of active commuting among children. As a result, these programs generally are not evidence-based and reflect assumed predictors of children's walking and bicycling to school. The research base has since expanded rapidly to enhance these programs. The effectiveness of future efforts to promote children's active commuting to school will be enhanced by drawing on the burgeoning research on predictors of active commuting to school, incorporating plans for evaluation into the initial planning stages of programs, and disseminating the results from formal and informal evaluations in outlets accessible to public health scientists and practitioners. Parental involvement is one example of how findings from research and evaluations of practice-based activities can be integrated. Research supports parents' roles as primary stakeholders in efforts to increase children's active commuting to school but often have little time to devote to programmatic activities. Consequently, although parental guidance and buy-in is necessary to the success of any program, programs that rely heavily on parent volunteers may be compromised in terms of their long-term feasibility. Other groups need to be considered to take on this role. For example, in the WSB program in Auckland, New Zealand, a retired volunteer helped supervise the walking routes (46). According to qualitative accounts, this was a role that the volunteer enjoyed, and his involvement facilitated continuity in the program (46). Efforts to integrate findings from research and evaluations of programs will facilitate identification of best practices for promoting active commuting and efficient use of limited resources.

## Future Directions

Increasing active commuting rates promises health benefits for future generations. In the short term, designing effective evidence-based programs will require high-quality research to accurately identify predictors of active commuting, including community, school, and family factors. Such efforts also should include an examination of school and regional policies and their effect on children's commuting patterns. In the long term, a continued research perspective will entail adequate monitoring of programs and programs for the desired outcome of more physically active children. The current federal program (SAFETEA-LU), which is expected to provide funds to build infrastructure and help develop effective programs, could provide a unique opportunity for preimplementation and postimplementation data collection and identification of successful strategies. The key is to develop standardized instruments and variable definitions to allow for comparative studies, provide a repository for the data (e.g., a secure Web site hosted by an academic institution), and develop a reporting platform that will make "lessons learned" available as quickly as possible. Such information could in turn be used to guide land use and transportation planners during consideration of changes in comprehensive planning programs, zoning regulations, and integration of walking patterns into engineering standards and to inform local planners and school district personnel when making decisions about school locations. Coordinating these short-term and long-term goals requires continued dialogue among public health professionals, local planners, and community members.
